# Association of visceral obesity indicators with prostate cancer: a cross-sectional study from Xinjiang

**DOI:** 10.3389/fonc.2025.1614743

**Published:** 2025-07-09

**Authors:** Zhiruo Cai, Xue Guan, Yunyun Xiao, Hengqing An, Ning Tao

**Affiliations:** ^1^ College of Public Health, Xinjiang Medical University, Urumqi, China; ^2^ Department of Urology, The First Affiliated Hospital of Xinjiang Medical University, Urumqi, China

**Keywords:** prostate cancer, visceral obesity indicators, cardiometabolic index, lipid accumulation product, visceral adiposity index

## Abstract

**Background:**

Prostate cancer (PCa) is a common malignancy among men worldwide, and its risk is strongly associated with obesity, especially visceral obesity. Visceral obesity has been assessed by the visceral adiposity index (VAI), cardiometabolic index (CMI), and lipid accumulation product (LAP), but their associations with PCa remain underexplored. This study investigated the relationship between these visceral obesity indicators and PCa risk.

**Methods:**

Data for this cross-sectional study were obtained from the First Affiliated Hospital of Xinjiang Medical University from 2022-2023, and 730 participants were screened for the study. A total of 102 PCa patients were included as the PCa group and 102 healthy individuals as the control group using propensity score matching (PSM). We collected anthropometric data (height, weight, waist circumference) and blood biochemical parameters from participants to calculate the VAI, CMI and LAP. These indicators’ association and predictive efficacy with PCa were assessed by logistic regression, restricted cubic spline (RCS), and receiver operating characteristic (ROC) curve analysis. The robustness of these results was further examined through sensitivity analyses.

**Results:**

VAI, CMI, and LAP were higher in the PCa group than in the control group (*P*<0.05). Logistic regression models showed that VAI, CMI, and LAP were positively associated with PCa. This association of VAI and CMI shows robustness in sensitivity analysis. Compared with the first quartile (Q1), the fourth quartile’s (Q4) VAI, CMI and LAP were linked to an increased risk of PCa (OR: 9.07, 95% CI: 3.21-25.65; OR: 11.10, 95% CI: 3.87-31.83; OR: 3.01, 95% CI: 1.17-7.76, respectively). RCS analysis showed that VAI and CMI were nonlinearly associated with PCa risk, and LAP was linearly associated with PCa risk. The area under the ROC curve (AUC) of VAI, CMI, and LAP was 0.721 (95% CI: 0.651-0.791), 0.711 (95% CI: 0.639-0.782), and 0.593 (95% CI: 0.515-0.671), respectively.

**Conclusions:**

Visceral obesity indicators are closely associated with PCa, of which VAI and CMI show good predictive value and robustness, and can be used as potential biomarkers for assessing PCa risk.

## 1 Introduction

In 2022, prostate cancer (PCa) ranks as the second most prevalent cancer globally (incidence 14.2%) and the fifth most common cause of cancer death (mortality 7.3%) among men (1). A new report in The Lancet estimates that annual new instances of PCa will increase from 1.4 million in 2020 to 2.9 million by 2040 (2). PCa is the most common cancer diagnosed in men over the age of 50 (3). In recent years, with the aging of the global population, the incidence of PCa has increased year by year (4). PCa has become a major public health problem for men worldwide and carries a huge economic burden (5).

Factors influencing PCa include age and genetics (6, 7), while modifiable exogenous factors such as diet, metabolic syndrome, and obesity are also involved in the development of PCa (8–10). Among these, the relationship between obesity and PCa is in the spotlight due to the rising number of obese persons worldwide (11). Obesity is closely related to tumor development, particularly visceral obesity, which is more likely to cause cancer than total body fat (12). There is ample evidence that visceral obesity is associated with poor prognosis and risk of recurrence in a variety of tumors (13–15). Visceral obesity is a state of excessive accumulation of visceral adipose tissue (VAT) in the abdominal cavity, which can reflect the distribution of fat (16). Excess visceral fat is associated with a higher risk of cardiometabolic disorders, including hypertension, cardiovascular disease, and dyslipidemia (17, 18). Evidence suggests that increased visceral obesity is connected with poor prognosis in PCa (19). In addition, periprostatic adipose tissue (PPAT), which is white visceral adipose tissue close to the prostate, is an important component of the PCa tumor microenvironment (20). Research has shown that PPAT is implicated in PCa development, progression, invasion, and metastasis through the release of numerous active molecules (20). Obesity-induced changes in PPAT gene expression promote PCa progression by stimulating cell proliferation and inhibiting immune surveillance (21).

Waist circumference (WC), body mass index (BMI), and waist-to-height ratio (WHtR) are widely employed as evaluation markers to define obesity, but these indicators don’t accurately reflect the body fat content, distribution, and function (22, 23). Currently, computed tomography (CT) or magnetic resonance imaging (MRI) can quantify visceral obesity (24, 25). However, imaging-based assessment of visceral obesity is unsuitable for mass screening, considering radiation exposure, operational complexity, and high economic expenditures. In recent years, researchers have proposed some novel indexes that can reflect visceral obesity and metabolic disorders: visceral adiposity index (VAI), cardiometabolic index (CMI), and lipid accumulation product (LAP) (26, 27). VAI is an essential indicator of visceral adipose tissue dysfunction and is associated with the risk of tumorigenesis (28). LAP reflects metabolic status and is an effective indicator for predicting cardiometabolic conditions (29). CMI, a novel indicator of visceral obesity, was initially used to predict diabetes (30). However, recent research has concluded that CMI is a more precise and refined indicator for identifying individuals at risk for cardiometabolic disease (31). These visceral obesity indicators combine anthropometric and lipid parameters, are easily available and more economical than traditional single obesity indicators, and help identify visceral fat dysfunction (32, 33).

Currently, research on visceral obesity indicators is mainly focused on the cardiovascular disease field, and its study in oncology is still in the emerging phase. Additionally, investigations of visceral obesity indicators in the context of PCa remain relatively limited. This study intends to explore the association between visceral obesity indicators and PCa, thereby filling the void in this field. Furthermore, it provides support for visceral obesity indicators as prospective biomarkers for PCa risk, providing greater knowledge of the risk factors for PCa.

## 2 Methods

### 2.1 Data source

Data for this study were retrieved from the database of the Urology and Physical Examination Center at the First Affiliated Hospital of Xinjiang Medical University, comprising all medical records of examinations performed at the Department of Urology and the Medical Examination Center between 2022 and 2023. All research participants willingly consented to participate and provided informed consent, and the study was authorized by the Medical Ethics Committee of the First Affiliated Hospital of Xinjiang Medical University (Approval number: XJYKDXR20240521004).

**Figure 1:**
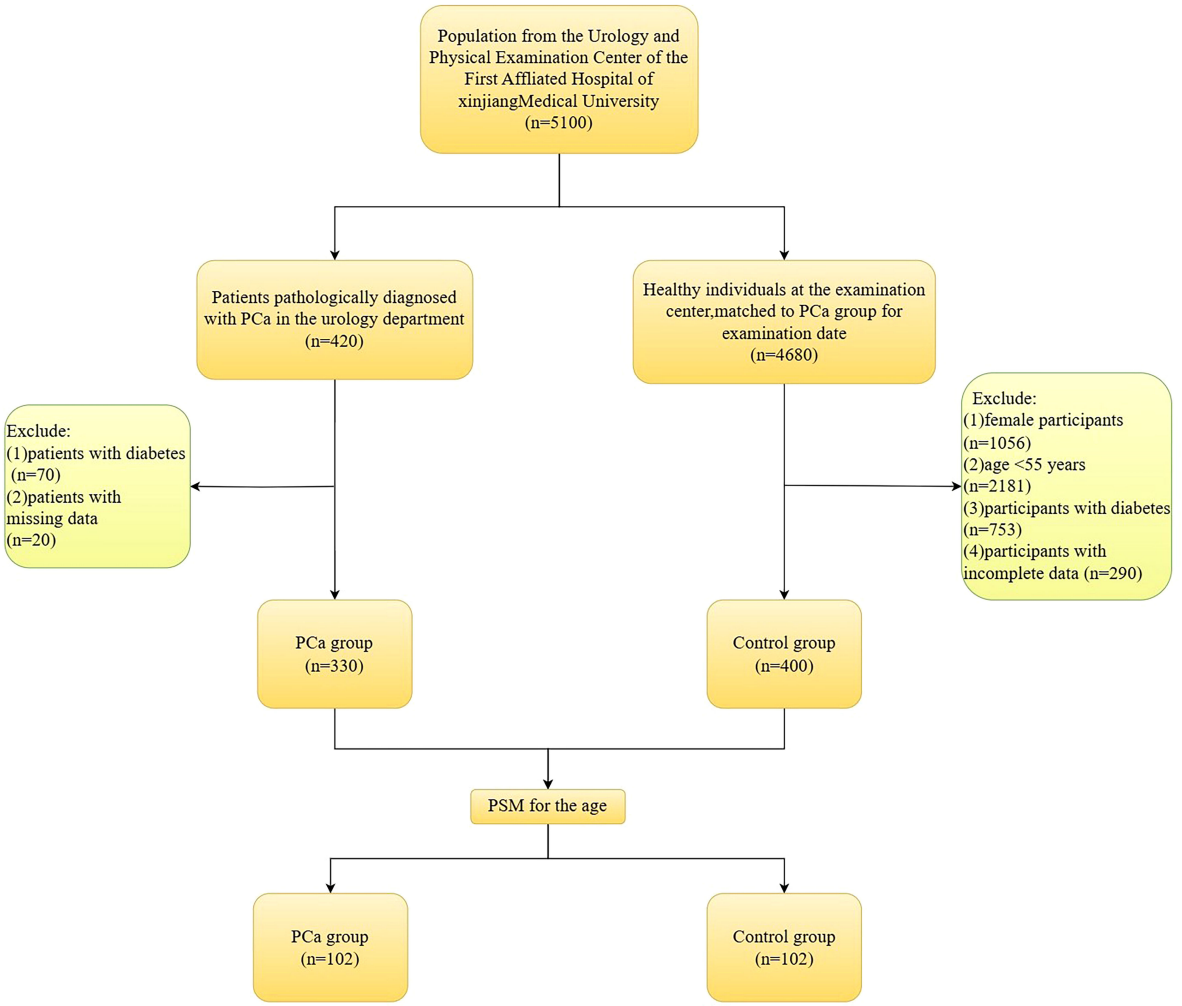
Flowchart of inclusion and exclusion of study participants.

This study included 5100 participants in total. These included 420 PCa patients who were pathologically diagnosed with PCa by prostate needle biopsies. Another 4,680 healthy individuals had a physical examination during the same period. Based on the inclusion and exclusion criteria, the study ultimately included 330 PCa patients and 400 non-PCa individuals. See **Figure 1**.

Inclusion criteria for the PCa group: (1) meeting the indications for prostate needle biopsy; (2) patients who were diagnosed with PCa based on the pathological assessment of their initial prostate needle biopsy; (3) participants who had signed consent forms; (4) those with complete data linked to this study. Exclusion criteria for the PCa group: (1) patients with diabetes; (2) individuals with missing data.

Inclusion criteria for the control group: (1) participants matched to the PCa group for examination date; (2) complete physical examination data; (3) participants who had signed consent forms. Exclusion criteria for the control group: (1) female participants; (2) age ≤55 years; (3) participants with diabetes; (4) participants with incomplete data.

This research used propensity score matching (PSM) based on age to account for its possible influence on PCa risk, resulting in the inclusion of 102 PCa group and 102 control group for analysis.

### 2.2 Data collection and measurements

The participants’ medical histories and physical examinations were obtained during the medical examination to gather their anthropometric measurements (height, weight, WC), as well as blood biochemistry markers. All participants underwent physical examinations in the morning in a fasted state. Physicians with specialized training measured anthropometric parameters. Anthropometric parameters were measured after participants stood naturally and removed their shoes and thick clothing. Participants’ height and weight were obtained using an ultrasonic height and weight measuring device, with a height accuracy of 0.1 cm. WC was measured with a soft tape at the midpoint between the iliac crest and the lower rib margin.

Blood samples were collected from the antecubital vessels of participants the following morning after they had fasted for a minimum of 8 hours. The hospital’s clinical laboratory received the blood samples for the analysis of triglycerides (TG), total cholesterol (TC), high-density lipoprotein cholesterol (HDL-C), blood urea nitrogen (BUN), serum uric acid (SUA), and creatinine (Cr) using a Dimension AR/AVL analyzer (Dade Behring, USA).

### 2.3 Calculation of visceral adiposity indicators


$$VAI=\left(\frac{WC}{39.68+1.88\times BMI}\right)\times \left(\frac{TG}{1.03}\right)\times \left(\frac{1.31}{\text{HDL}-\text{C}}\right)$$

(34)


$$CMI=\left(\frac{TG}{HDL-C}\right)\times \left(\frac{WC}{Height}\right)$$

(30)


LAP=(WC−65)×TG

(35)

### 2.4 Statistical analysis

Statistical analyses were performed using SPSS version 25.0 and R (version 4.4.1). Normally distributed measurement data were presented as mean ± standard deviation (SD) and compared by *t*-tests. Non-normally distributed measurement data were presented as median and interquartile range and examined with non-parametric tests. Categorical variables were presented as percentage frequencies (%). Differences between groups were assessed using the *χ²* test. PSM was used to balance the age difference between the two research groups using the closest neighbor matching technique (1:1) and the caliper value (0.02) with a two-sided test level of *α* < 0.05. The correlation between visceral obesity indicators and PCa was examined by logistic regression, both before and after PSM. Restricted cubic spline (RCS) models were used to analyze the dose-response relationship between visceral obesity indicators and PCa risk, and the difference was statistically significant at *α* < 0.05. Receiver operating characteristic (ROC) curve analysis was used to assess the predictive performance of visceral adiposity indicators for PCa risk. After removing outliers, sensitivity analyses were performed using logistic regression and Poisson regression to evaluate the stability of the results.

## 3 Results

### 3.1 Characteristics of Lipid and visceral obesity indicators in participants, and their association with PCa risk before PSM

Before PSM, a total of 730 people were enrolled in the study, 330 in the PCa group and 400 in the control group. There were statistically significant differences in Age, TG, HDL-C, TC, VAI, CMI, LAP, Cr, and SUA (*P*< 0.05) between the two groups. There was no statistically significant difference between the two groups in terms of BUN (*P* > 0.05). See **Table 1**. Compared with the lowest VAI, CMI, and LAP quartile, the OR (95% CI) for PCa prevalence in the highest quartiles was 11.47(95% CI 6.47-20.35), 8.74(5.03-15.17), 4.23 (2.52-7.12), respectively. See **Table 2**.

**Table 1:** Characterization of blood biochemical indices and visceral obesity indicators in PCa group and control group before PSM.

Variables	Total (n = 730)	Control group (n = 400)	PCa group (n = 330)	*t/Z/χ²*	*P*
Age, M (Q_1_, Q_3_) (year)	80.00 (70.00,84.00)	83.00 (81.00,86.00)	68.00(62.00,76.00)	25.00	<0.001
TG (mmol/L)	1.41 ± 0.73	1.29 ± 0.66	1.56 ± 0.79	-5.05	<0.001
HDL-C (mmol/L)	1.16 ± 0.31	1.24 ± 0.29	1.06 ± 0.30	8.16	<0.001
TC (mmol/L)	4.50 ± 1.11	4.76 ± 1.13	4.17 ± 0.99	7.50	<0.001
VAI	128.32 ± 92.20	107.15 ± 75.91	153.98 ± 103.16	-6.86	<0.001
CMI	0.73 ± 0.50	0.62 ± 0.39	0.87 ± 0.58	-6.87	<0.001
LAP	42.19 ± 29.48	38.20 ± 23.28	47.02 ± 35.02	-3.92	<0.001
BUN, M (Q_1_, Q_3_) (mmol/L)	6.22 (5.10, 7.46)	6.26 (5.15, 7.50)	6.19 (4.96, 7.34)	-1.16	0.247
Cr, M (Q_1_, Q_3_) (μmol/L)	79.65 (67.51, 93.07)	83.31 (71.10, 96.28)	74.31 (62.36, 88.00)	-6.25	<0.001
SUA, M (Q_1_, Q_3_) (μmol/L)	324.20 (276.40, 392.00)	335.50 (291.00, 396.49)	306.30 (255.00, 376.98)	-4.21	<0.001
VAI, n (%)				63.94	<0.001
Q1(<65.11)	183 (25.07)	141 (35.25)	42 (12.73)		
Q2(65.11-104.60)	182 (24.93)	104 (26.00)	78 (23.64)		
Q3(104.60-164.16)	181 (24.79)	87 (21.75)	94 (28.48)		
Q4(≥164.16)	184 (25.21)	68 (17.00)	116 (35.15)		
CMI, n (%)				48.47	<0.001
Q1(<0.38)	183 (25.07)	133 (33.25)	50 (15.15)		
Q2(0.38-0.59)	181 (24.79)	107 (26.75)	74 (22.42)		
Q3(0.59-0.92)	183 (25.07)	91 (22.75)	92 (27.88)		
Q4(≥0.92)	183 (25.07)	69 (17.25)	114 (34.55)		
LAP, n (%)				12.69	0.005
Q1(<23.35)	182 (24.93)	116 (29.00)	66 (20.00)		
Q2(23.35-35.89)	183 (25.07)	103 (25.75)	80 (24.24)		
Q3(35.89-54.74)	182 (24.93)	98 (24.50)	84 (25.45)		
Q4(≥54.74)	183 (25.07)	83 (20.75)	100 (30.30)		

TG, triglycerides; HDL-C, high-density lipoprotein cholesterol; TC, total cholesterol; VAI, visceral adiposity index; CMI, cardiometabolic index; LAP, lipid accumulation product; SUA, Serum uric acid; BUN, blood urea nitrogen; Cr, Creatinine.

**Table 2:** The association between visceral obesity indicators and Pca risk: univariable and multivariable conditional logistic regression analyses before PSM.

Variables	Univariate conditional logistic regression analysis					Multivariate conditional logistic regression analysis				
	β	S.E	Z	*P*	OR (95%CI)	β	S.E	Z	*P*	OR (95%CI)
VAI	0.01	0.001	6.383	<0.001	1.01 (1.004 ~ 1.01)	0.01	0.001	7.29	<0.001	1.01 (1.006 ~ 1.01)
VAI(Q4)										
Q1(<65.11)					1.00 (Reference)					1.00 (Reference)
Q2(65.11-104.60)	0.92	0.23	4.00	<0.001	2.52 (1.60 ~ 3.96)	1.39	0.28	4.92	<0.001	4.03 (2.31 ~ 7.01)
Q3(104.60-164.16)	1.29	0.23	5.59	<0.001	3.63 (2.31 ~ 5.70)	1.71	0.28	6.07	<0.001	5.53 (3.18 ~ 9.61)
Q4(≥164.16)	1.75	0.23	7.49	<0.001	5.73 (3.63 ~ 9.04)	2.44	0.29	8.35	<0.001	11.47 (6.47 ~ 20.35)
CMI	1.16	0.18	6.43	<0.001	3.17 (2.23 ~ 4.51)	1.65	0.22	7.53	<0.001	5.22 (3.39 ~ 8.02)
CMI(Q4)										
Q1(<0.38)					1.00 (Reference)					1.00 (Reference)
Q2(0.38-0.59)	0.61	0.22	2.72	<0.001	1.84 (1.18 ~ 2.86)	0.94	0.27	3.46	<0.001	2.56 (1.50 ~ 4.36)
Q3(0.59-0.92)	0.99	0.22	4.45	<0.001	2.69 (1.74 ~ 4.16)	1.32	0.27	4.81	<0.001	3.73 (2.18 ~ 6.37)
Q4(≥0.92)	1.48	0.23	6.57	<0.001	4.39 (2.83 ~ 6.84)	2.17	0.28	7.70	<0.001	8.74 (5.03 ~ 15.17)
LAP	0.01	0.003	3.86	<0.001	1.01 (1.005 ~ 1.02)	0.02	0.004	5.64	<0.001	1.02 (1.01 ~ 1.03)
LAP(Q4)										
Q1(<23.35)					1.00 (Reference)					1.00 (Reference)
Q2(23.35-35.89)	0.31	0.21	1.45	0.147	1.37 (0.90 ~ 2.08)	0.46	0.26	1.74	0.082	1.58 (0.94 ~ 2.63)
Q3(35.89-54.74)	0.41	0.21	1.91	0.056	1.51 (0.99 ~ 2.29)	0.93	0.26	3.50	<0.001	2.52 (1.50 ~ 4.24)
Q4(≥54.74)	0.75	0.21	3.50	<0.001	2.12 (1.39 ~ 3.22)	1.44	0.27	5.44	<0.001	4.23 (2.52 ~ 7.12)

VAI, visceral adiposity index; CMI, cardiometabolic index; LAP, lipid accumulation product; OR, Odds ratio.

### 3.2 Characteristics of lipid and visceral obesity indicators in participants, and their association with PCa risk after PSM

As preliminary analyses showed differences in age between the two groups, PSM (1:1 precise age matching) was performed in this study to ensure the two groups were comparable. In logistic regression analyses, VAI, CMI and LAP expressed as either continuous variables or quartiles. Multivariable logistic regression demonstrates that higher VAI, CMI, and LAP levels were independently associated with increased PCa odds. Compared with the lowest quartile (Q1) of VAI, the OR (95% CI) for the PCa risk in the second, third, and fourth quartiles of VAI was 3.44 (1.25-9.46), 8.76 (3.12-24.62) and 9.07 (3.21-25.65), respectively. Compared with the lowest quartile (Q1) of CMI, the OR (95% CI) for the PCa risk in the second, third, and fourth quartiles of CMI was 4.69 (1.67-13.20), 7.10 (2.58-19.54) and 11.10 (3.87-31.83), respectively. The OR (95% CI) values of LAP in the third and fourth quartile groups were 3.01 (1.16-7.83) and 3.01 (1.17-7.76), respectively, using the lowest quartile group as a reference. See **Tables 3**, **4**.

**Table 3:** Characterization of blood biochemical indices and visceral obesity indicators in PCa group and control group after PSM.

Variables	Total (n = 204)	PCa group (n = 102)	Control group (n = 102)	*t/Z/χ²*	*P*
Age, Mean ± SD (year)	77.35 ± 6.27	76.94 ± 6.65	77.76 ± 5.88	0.94	0.350
TG (mmol/L)	1.39 ± 0.71	1.41 ± 0.71	1.37 ± 0.71	-0.39	0.698
HDL-C (mmol/L)	1.15 ± 0.30	1.07 ± 0.29	1.22 ± 0.29	3.81	<0.001
TC (mmol/L)	4.36 ± 1.13	4.05 ± 1.02	4.67 ± 1.16	4.07	<0.001
VAI, M (Q_1_, Q_3_)	83.14 (52.02, 149.09)	107.99 (71.79, 175.11)	60.65 (41.04, 105.36)	-5.45	<0.001
CMI, M (Q_1_, Q_3_)	0.50 (0.30, 0.88)	0.60 (0.41, 0.92)	0.35 (0.24, 0.58)	-5.20	<0.001
LAP, M (Q_1_, Q_3_)	29.07 (17.55, 45.54)	107.99 (71.79, 175.11)	25.21 (14.31, 41.14)	-2.30	0.022
BUN, M (Q_1_, Q_3_) (mmol/L)	6.16 (4.80, 7.22)	6.40 (4.80, 7.35)	5.68 (4.98, 7.06)	-1.03	0.301
Cr, M (Q_1_, Q_3_) (μmol/L)	79.90 (68.72, 95.09)	75.00 (62.00, 94.66)	83.30 (73.65, 95.27)	-2.69	0.007
SUA, M (Q_1_, Q_3_) (μmol/L)	324.20 (272.08, 376.98)	305.75 (254.08, 376.20)	333.26 (284.35, 382.38)	-1.66	0.097
VAI, n (%)				63.94	<0.001
Q1(<51.92)	183 (25.07)	141 (35.25)	42 (12.73)		
Q2(51.92-83.14)	182 (24.93)	104 (26.00)	78 (23.64)		
Q3(104.60-149.10)	181 (24.79)	87 (21.75)	94 (28.48)		
Q4(≥149.10)	184 (25.21)	68 (17.00)	116 (35.15)		
CMI, n (%)				48.47	<0.001
Q1(<0.30)	183 (25.07)	133 (33.25)	50 (15.15)		
Q2(0.30-0.50)	181 (24.79)	107 (26.75)	74 (22.42)		
Q3(0.50-0.89)	183 (25.07)	91 (22.75)	92 (27.88)		
Q4(≥0.89)	183 (25.07)	69 (17.25)	114 (34.55)		
LAP, n (%)				12.69	0.005
Q1(<17.50)	182 (24.93)	116 (29.00)	66 (20.00)		
Q2(17.50-29.07)	183 (25.07)	103 (25.75)	80 (24.24)		
Q3(29.07-45.62)	182 (24.93)	98 (24.50)	84 (25.45)		
Q4(≥45.62)	183 (25.07)	83 (20.75)	100 (30.30)		

VAI, visceral adiposity index; CMI, cardiometabolic index; LAP, lipid accumulation product.

**Table 4:** The association between visceral obesity indicators and Pca risk: univariable and multivariable logistic regression analyses after PSM.

Variables	Univariate conditional logistic regression analysis					Multivariate conditional logistic regression analysis				
	β	S.E	Z	*P*	OR (95%CI)	β	S.E	Z	*P*	OR (95%CI)
VAI	0.01	0.00	3.36	<0.001	1.01 (1.01 ~ 1.01)	1.41	0.43	3.31	<0.001	4.10 (1.78 ~ 9.47)
VAI(Q4)										
Q1(<51.92)					1.00 (Reference)					1.00 (Reference)
Q2(51.92-83.14)	1.42	0.40	3.59	<0.001	4.13 (1.90 ~ 8.95)	1.24	0.52	2.40	0.016	3.44 (1.25 ~ 9.46)
Q3(104.60-149.10)	1.65	0.44	3.78	<0.001	5.21 (2.21 ~ 12.28)	2.17	0.53	4.12	<0.001	8.76 (3.12 ~ 24.62)
Q4(≥149.10)	1.98	0.42	4.69	<0.001	7.26 (3.17 ~ 16.61)	2.20	0.53	4.16	<0.001	9.07 (3.21 ~ 25.65)
CMI	1.44	0.40	3.62	<0.001	4.20 (1.93 ~ 9.13)	0.93	0.26	3.50	<0.001	2.52 (1.50 ~ 4.24)
CMI(Q4)										
Q1(<0.30)					1.00 (Reference)					1.00 (Reference)
Q2(0.30-0.50)	1.17	0.39	3.03	0.002	3.22 (1.51 ~ 6.87)	1.55	0.53	2.93	0.003	4.69 (1.67 ~ 13.20)
Q3(0.50-0.89)	1.60	0.42	3.81	<0.001	4.96 (2.18 ~ 11.29)	1.96	0.52	3.79	<0.001	7.10 (2.58 ~ 19.54)
Q4(≥0.89)	1.72	0.43	4.03	<0.001	5.58 (2.42 ~ 12.85)	2.41	0.54	4.48	<0.001	11.10 (3.87 ~ 31.83)
LAP	0.01	0.01	1.73	0.084	1.01 (1.00 ~ 1.02)	0.02	0.01	2.14	0.033	1.02 (1.01 ~ 1.03)
LAP(Q4)										
Q1(<17.50)					1.00 (Reference)					1.00 (Reference)
Q2(17.50-29.07)	0.27	0.37	0.74	0.462	1.31 (0.64 ~ 2.68)	0.51	0.48	1.06	0.291	1.66 (0.65 ~ 4.27)
Q3(29.07-45.62)	0.52	0.40	1.31	0.191	1.68 (0.77 ~ 3.68)	1.10	0.49	2.26	0.024	3.01 (1.16 ~ 7.83)
Q4(≥45.62)	0.78	0.40	1.95	0.052	2.18 (0.99 ~ 4.79)	1.10	0.48	2.28	0.023	3.01 (1.17 ~ 7.76)

VAI, visceral adiposity index; CMI, cardiometabolic index; LAP, lipid accumulation product.

### 3.3 Dose-response relationship between visceral obesity indicators and PCa

**Figure 2:**
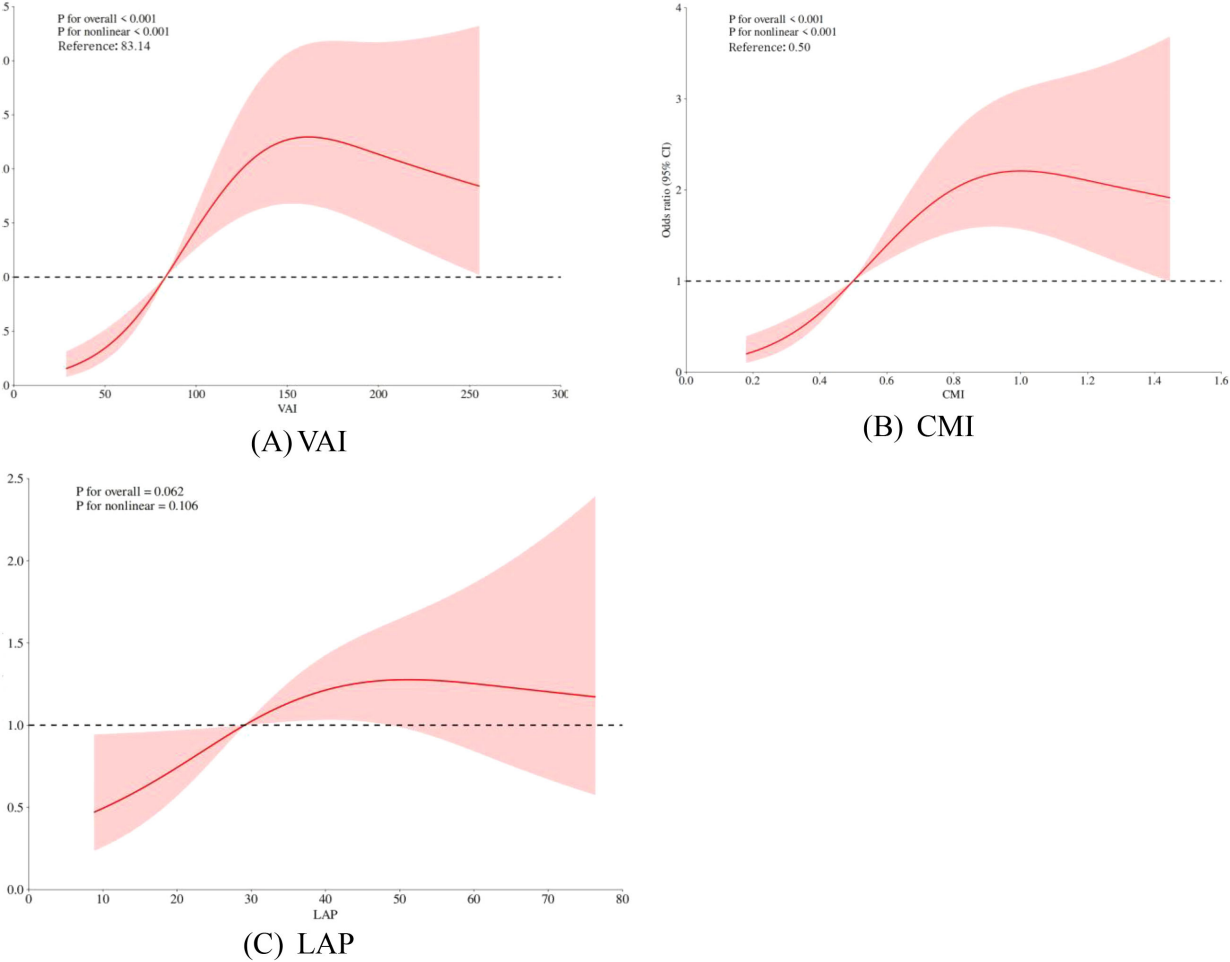
**(A-C)** Dose-response relationship between VAI, CMI, LAP and PCa prevalence.

Restricted cubic spline (RCS) models were used to analyze further the association between visceral obesity indices and PCa. The results showed that VAI and CMI exhibited a non-linear dose-response relationship with PCa risk (*P* for overall < 0.001, *P* for nonlinear < 0.05), while LAP exhibited a linear dose-response relationship with PCa risk (*P* for nonlinear > 0.05). See **Figures 2A–C**.

The red solid line represents the odds ratio (OR), the red shaded area indicates the 95% confidence intervals (95% CI), and the horizontal dashed line marks the null line (OR = 1).

### 3.4 The predictive value of visceral obesity indicators in PCa risk assessment

**Figure 3:**
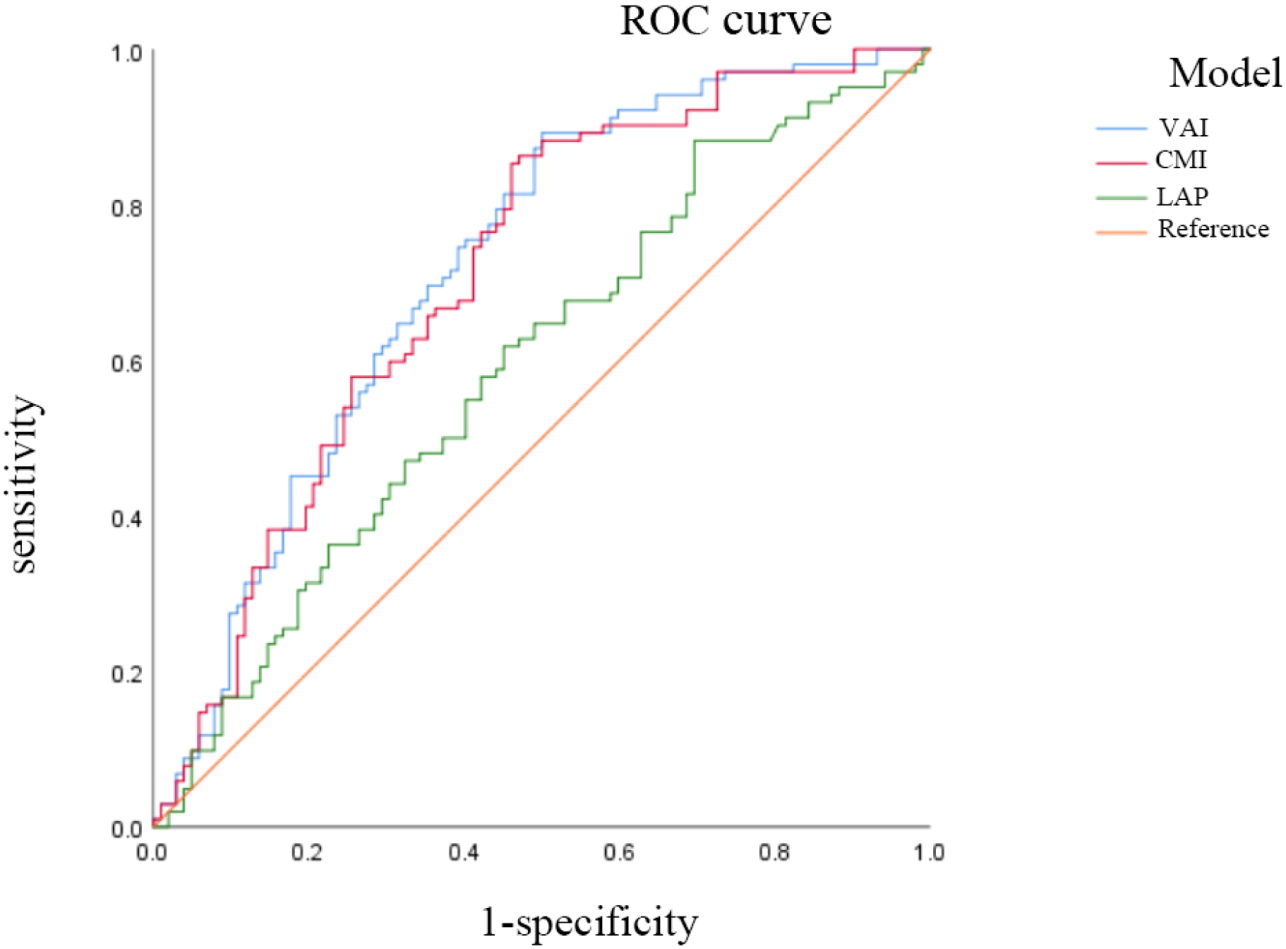
The ROC analysis of VAI, CMI and LAP on PCa. AUC: the area under the curve. ratio. VAI, visceral adiposity index; CMI, cardiometabolic index; LAP, lipid accumulation product.

ROC analysis demonstrated distinct predictive capacities among the visceral obesity indicators. The area under curve (AUC) of VAI, CMI, and LAP were 0.721 (95% CI: 0.651-0.791), 0.711 (95% CI: 0.639-0.782), and 0.593 (95% CI: 0.515-0.671), respectively. VAI had a relatively high predictive ability for PCa risk. See **Figure 3**.

### 3.5 Sensitivity analysis of visceral obesity indicators and PCa risk

The results revealed that the association between VAI, CMI, and PCa remained stable in both logistic regression and Poisson regression models. Treating VAI and CMI as categorical variables still yielded the conclusion that there is a positive correlation between VAI, CMI and the odds of PCa prevalence. Participants with higher VAI, CMI had higher odds of PCa, further increasing the reliability of our study conclusions. See **Tables 5** and **6**.

**Table 5:** Sensitive analysis on the association between visceral obesity indicators and PCa using logistic regression.

Variables	Univariate conditional logistic regression analysis					Multivariate conditional logistic regression analysis				
	β	S.E	Z	*P*	OR (95%CI)	β	S.E	Z	*P*	OR (95%CI)
VAI	0.01	0.00	3.90	<0.001	1.01 (1.01 ~ 1.02)	0.01	0.00	3.29	<0.001	1.01 (1.01 ~ 1.02)
VAI(Q4)										
Q1(<65.19)					1.00 (Reference)					1.00 (Reference)
Q2(65.19-104.60)	1.45	0.46	3.14	0.002	4.27 (1.72 ~ 10.59)	1.12	0.52	2.15	0.031	3.06 (1.11 ~ 8.48)
Q3(104.60-164.06)	2.07	0.47	4.38	<0.001	7.90 (3.13 ~ 19.93)	2.03	0.53	3.85	<0.001	7.64 (2.71 ~ 21.53)
Q4(≥164.06)	2.35	0.49	4.82	<0.001	10.48 (4.03 ~ 27.25)	2.10	0.54	3.86	<0.001	8.15 (2.81 ~ 23.63)
CMI	1.87	0.47	4.02	<0.001	6.49 (2.61 ~ 16.15)	1.79	0.52	3.46	<0.001	6.00 (2.17 ~ 16.56)
CMI(Q4)										
Q1(<0.38)					1.00 (Reference)					1.00 (Reference)
Q2(0.38-0.59)	1.36	0.45	3.00	0.003	3.90 (1.60 ~ 9.49)	1.45	0.53	2.74	0.006	4.27 (1.51 ~ 12.08)
Q3(0.59-0.92)	1.75	0.45	3.86	<0.001	5.76 (2.37 ~ 14.00)	1.84	0.52	3.55	<0.001	6.30 (2.28 ~ 17.40)
Q4(≥0.92)	2.29	0.48	4.75	<0.001	9.90 (3.84 ~ 25.49)	2.32	0.55	4.22	<0.001	10.19 (3.47 ~ 29.92)
LAP	0.01	0.01	1.97	0.049	1.01 (1.01 ~ 1.03)	0.02	0.01	1.99	0.047	1.02 (1.01 ~ 1.03)
LAP(Q4)										
Q1(<23.38)					1.00 (Reference)					1.00 (Reference)
Q2(23.38-35.89)	0.34	0.41	0.84	0.404	1.40 (0.63 ~ 3.11)	0.34	0.49	0.69	0.492	1.40 (0.54 ~ 3.65)
Q3(35.89-54.60)	0.65	0.41	1.61	0.108	1.92 (0.87 ~ 4.26)	0.94	0.49	1.90	0.057	2.55 (0.97 ~ 6.68)
Q4(≥54.60)	0.90	0.42	2.13	0.033	2.46 (1.08 ~ 5.61)	0.92	0.50	1.86	0.064	2.52 (0.95 ~ 6.70)

VAI, visceral adiposity index; CMI, cardiometabolic index; LAP, lipid accumulation product.

**Table 6:** Sensitive analysis on the association between visceral obesity indicators and PCa using oisson regression. .

Variables	Univariate conditional Poisson regression analysis					Multivariate conditional Poisson regression analysis				
	β	S.E	Z	*P*	OR (95%CI)	β	S.E	Z	*P*	OR (95%CI)
VAI	0.002	0.001	2.54	<0.05	1.002 (1.00~ 1.004)	0.002	0.001	2.36	<0.05	1.002 (1.00 ~ 1.004)
VAI(Q4)										
Q1(<65.19)					1.00 (Reference)					1.00 (Reference)
Q2(65.19-104.60)	1.02	0.39	2.63	<0.05	2.78(1.35 ~ 6.29)	0.74	0.40	1.86	0.06	2.10 (0.99 ~ 4.82)
Q3(104.60-164.06)	1.29	0.38	3.42	<0.001	3.63 (1.81 ~ 8.08)	1.10	0.38	2.97	<0.05	2.99(1.47 ~ 6.71)
Q4(≥164.06)	1.37	0.37	3.67	<0.001	3.92 (1.98 ~ 8.68)	1.11	0.38	2.93	<0.05	3.02(1.51~ 6.74)
CMI	0.47	0.17	2.72	<.05	1.60 (1.12 ~ 2.21)	0.45	0.19	2.39	<0.05	1.56 (1.06 ~ 2.22)
CMI(Q4)										
Q1(<0.38)					1.00 (Reference)					1.00 (Reference)
Q2(0.38-0.59)	0.94	0.37	2.50	0.01	2.55 (1.27 ~ 5.57)	0.85	0.38	2.23	0.03	2.34 (1.36 ~ 5.17)
Q3(0.59-0.92)	1.11	0.36	3.06	<0.05	3.04 (1.55~6.53)	0.98	0.37	2.69	0.001	2.67 (1.35 ~ 5.75)
Q4(≥0.92)	1.28	0.36	3.58	<0.001	3.60 (1.86 ~ 7.66)	1.11	0.36	3.07	<0.001	3.02 (1.55 ~ 6.46)
LAP	0.005	0.004	1.24	0.22	1.01 (0.99~ 1.01)	0.01	0.48	0.60	0.55	1.01 (1.00 ~ 1.02)
LAP(Q4)										
Q1(<23.38)					1.00 (Reference)					1.00 (Reference)
Q2(23.38-35.89)	0.23	0.31	0.76	0.45	1.26(0.69 ~ 2.33)	0.31	0.33	0.92	0.36	1.36 (0.72 ~ 2.65)
Q3(35.89-54.60)	0.39	0.30	1.31	0.19	1.47 (0.83 ~ 2.68)	0.49	0.32	1.52	0.13	1.63 (0.88 ~ 3.12)
Q4(≥54.60)	0.49	0.29	1.68	0.09	1.63 (0.93 ~ 2.94)	0.50	0.31	1.63	0.10	1.65 (0.92~ 3.11)

VAI, visceral adiposity index; CMI, cardiometabolic index; LAP, lipid accumulation product.

## 4 Discussion

The majority of earlier research has focused on the relationship between overall obesity and PCa (36, 37). Recent studies using imaging techniques to quantify visceral obesity have shown a significant correlation with an increased risk of PCa (14, 38). Visceral obesity, accompanied by dyslipidemia, insulin resistance, increased oxidative stress, and chronic inflammation, is the main mechanism of tumor development. In our study, we used a relatively rapid and cost-effective indicator of visceral obesity to explore the association between visceral obesity and PCa risk, thereby providing new insights into the intrinsic link between PCa and visceral obesity. Existing research indicates that VAI exhibits a positive association with insulin, glucose, and tumor necrosis factor-alpha (TNF-α) and demonstrates a negative association with the adiponectin: leptin ratio (39). This suggests that visceral obesity is tightly connected to metabolic dysregulation and inflammatory responses, which may collectively promote the development and progression of PCa.

Visceral obesity is commonly accompanied by dyslipidemia, typically presenting as increased triglycerides (TG) and low-density lipoprotein cholesterol (LDL-C), along with decreased high-density lipoprotein cholesterol (HDL-C) (40). Prior research has established a link between visceral obesity indicators and a range of metabolic disorders, including atherosclerosis, metabolic syndrome, hyperuricemia, and hypertension (41–43). Studies of lipid-related risk factors for PCa have shown that elevated TG levels are associated with increased risk and severity of PCa (44). Furthermore, TG and HDL-C levels are also recognized as significant factors linked to higher PCa prevalence (6). Consistent with the previous findings, our study reveals that the PCa group had higher blood lipid levels than the control group. Meanwhile, its visceral obesity indicators are also higher than those of the control group. This is because the calculation of visceral obesity indicators incorporates TG and HDL-C, allowing these indicators to comprehensively reflect visceral fat accumulation and related lipid metabolic abnormalities, highlighting their potential value in PCa risk assessment. Low HDL-C levels are the most common type of dyslipidemia in the middle-aged and elderly population in China (45), and the calculation of LAP, which involves only WC and TG, may not adequately capture the risk posed by reduced HDL-C. Besides, studies have revealed that lower levels of HDL-C may be connected with an increased risk of PCa (46). The anti-inflammatory and antioxidant capabilities of HDL-C may limit the development and progression activities of PCa cells (46). In contrast, other visceral obesity indicators, such as VAI and CMI, may contain more information about fat distribution and lipid metabolism and thus show greater robustness in sensitivity analyses. Therefore, although LAP showed some potential value in this study, its ability to predict PCa risk was inadequate.

In our study, we recognized a positive correlation between visceral obesity indicators and PCa. Moreover, VAI and CMI, which are indicators of visceral obesity, were nonlinearly associated with PCa risk. The storage capacity of visceral fat is maintained in a controllable range when the levels of VAI and CMI are low (VAI<83.14, CMI<0.50). Compensatory mechanisms, such as adipocyte remodeling and inflammation regulation, can effectively maintain the body’s balance (47), resulting in a gradual increase in PCa risk. Nevertheless, the aforementioned compensatory mechanisms are rendered ineffective when VAI and CMI surpass the threshold (VAI>83.14, CMI>0.50), resulting in the overexpression of inflammatory cytokines and adipocyte dysfunction, which contribute to an accelerated increase in PCa risk. The reason for these results may be that, in the obese condition, visceral adipose tissue can influence the development of PCa by releasing more adipokines (48). Visceral obesity leads to dysfunction of visceral adipose tissue, causing elevated triglyceride levels, which may be a potential mechanism by which adipose tissue contributes to PCa progression (49). Excess triglycerides mean increased free fatty acids in the blood, consequently resulting in elevated reactive oxygen radicals and oxidative stress (50, 51). Oxidative stress can directly damage cellular DNA, increasing the risk of cellular carcinogenesis (52). Reactive oxygen radicals can also activate mitogen-activated protein kinase (MAPK) and nuclear factor kappa-B (NF-κB) pathways to promote tumor growth and metastasis (53). In addition, insulin resistance develops as a result of increased free fatty acids interfering with normal insulin signal transduction (54). Insulin resistance leads to elevated insulin levels in the blood. This activates the insulin/insulin-like growth factor-1 (IGF-1) axis and subsequently promotes PCa cell proliferation and survival through signaling pathways such as phosphatidylinositol 3-kinase (PI3K)/protein kinase B (Akt) and MAPK (55, 56). Therefore, early intervention at important inflection points of VAI and CMI to minimize PCa risk is required.

Notably, CMI, a novel measure of visceral obesity, was much more strongly associated with PCa risk than VAI and LAP. After propensity scoring, there was a 3.20-fold increase in PCa risk for each unit increase in CMI. CMI calculation integrates waist circumference, height, and metabolic indicators (HDL-C and TG), thus providing a comprehensive assessment of visceral adiposity and metabolic health (34, 57). In contrast, the calculation of LAP only considers waist circumference and TG; its ability to comprehensively assess may be limited. Studies demonstrate that CMI can reflect systemic inflammatory states (58). Chronic low-grade inflammation due to obesity is associated with PCa, and visceral adipose tissue is a major source of chronic inflammation in the obese state (59). As weight increases, when lipid accumulation exceeds the storage capacity of subcutaneous fat, lipids accumulate in visceral adipose, causing adipocyte hypertrophy, hypoxia, and eventual cell death (60, 61). In the process, adipocytes release chemokines and recruit immune cells, driving the production of pro-inflammatory cytokines that promote chronic inflammation (20, 62). Mounting evidence suggests that multiple chemokines play a crucial role in obesity-driven PCa progression. Under obese conditions, increased CXC chemokine ligand 1 (CXCL1) expression is associated with PCa aggressiveness (63); elevated CXC chemokine ligand 8 (CXCL8) and CXC chemokine ligand 12 (CXCL12) expression are positively correlated with PCa metastasis (64–66). Additionally, mature adipocytes can secrete CC-chemokine ligand 7 (CCL7), which directly promotes cancer cell migration by interacting with the CC-chemokine receptor 3 (CCR3) on PCa cells (67). In white adipose tissue, which contains the highest proportion of adipose tissue, macrophages are the predominant immune cells, and their numbers increase with the progression of obesity (68). In murine models of obesity, the number of macrophages in white adipose tissue increases, accompanied by a corresponding elevation in the secretion of pro-inflammatory cytokines (66).

In summary, visceral obesity, as a modifiable risk factor, plays a significant role in reducing the risk of PCa through early identification and effective management. This study provides new evidence for the association between visceral obesity and PCa using a relatively rapid and cost-effective indicator of visceral obesity. These findings lay a certain foundation for early screening and risk assessment of PCa and provide new clues for further investigation into the etiology of the disease.

## 5 Limitations

Firstly, this is a cross-sectional study, and the causal relationship between VAI, CMI, LAP, and PCa could not be analyzed. Therefore, further prospective studies are needed to determine the exact relationship between the visceral obesity indicators and the PCa Risk. Secondly, given the data are sourced from a single institution and the relatively small sample size, which limits the extrapolation of results to a larger population. Multicenter studies are still needed in the future to include more participants and validate the generalizability of the research findings. Thirdly, other factors that may affect the study results, such as lifestyle factors, were not considered in this study. Future studies should strive to account for these factors to reduce potential bias and more accurately assess the independent relationship between visceral obesity and PCa risk.

## 6 Conclusions

This study demonstrates that VAI, CMI, and LAP are significantly associated with the risk of developing PCa. Elevated levels of VAI and CMI correspond to an increased risk of PCa, with this association demonstrating notable robustness in sensitivity analyses. RCS analysis revealed a non-linear relationship between VAI and CMI with PCa risk, while LAP exhibited a linear relationship. These results further reveal the significant role of visceral obesity indicators in the pathogenesis of PCa.

## Data Availability

The data analyzed in this study is subject to the following licenses/restrictions: The data that support the findings of this study are available from the corresponding author upon reasonable request. Requests to access these datasets should be directed to NT 38518412@qq.com.
